# Supplementary material for: Anti-HIV Potential of Beesioside I Derivatives as Maturation Inhibitors: Synthesis, 3D-QSAR, Molecular Docking and Molecular Dynamics Simulations
Source: Int J Mol Sci. 2023 Jan 11;24(2):1430. doi: 10.3390/ijms24021430 (PMC9867151; doi:10.3390/ijms24021430)
Supplement: Supplementary file 1 [file ijms-24-01430-s001.zip › ijms-2119651-supplementary.pdf]

Supplementary Materials

Table of Contents

Figure S1. Kinetic, potential energy variations and temperature changes in the MD simulations of BVM (**a**) and DSC (**b**).

Figure S2. Total energy frequency distribution of BVM **(a)** and DSC **(b)**.

Figure S3. Lowest potential energy structure of BVM **(a)** and DSC **(b)**.

Figure S4. The ^{1}H NMR (600 MHz, pyridine-*d*$_5$) spectrum of the new derivative **2a**

Figure S5. The ^{13}C NMR (150 MHz, pyridine-*d*$_5$) spectrum of the new derivative **2a**

Figure S6. The HRESIMS spectrum of the new derivative **2a**

Figure S7. The ^{1}H NMR (600 MHz, pyridine-*d*$_5$) spectrum of the new derivative **2b**

Figure S8. The ^{13}C NMR (150 MHz, pyridine-*d*$_5$) spectrum of the new derivative **2b**

Figure S9. The HRESIMS spectrum of the new derivative **2b**

Figure S10. The ^{1}H NMR (600 MHz, pyridine-*d*$_5$) spectrum of the new derivative **3a**

Figure S11. The ^{13}C NMR (150 MHz, pyridine-*d*$_5$) spectrum of the new derivative **3a**

Figure S12. The HRESIMS spectrum of the new derivative **3a**

Figure S13. The ^{1}H NMR (600 MHz, pyridine-*d*$_5$) spectrum of the new derivative **3b**

Figure S14. The ^{13}C NMR (150 MHz, pyridine-*d*$_5$) spectrum of the new derivative **3b**

Figure S15. The HRESIMS spectrum of the new derivative **3b**

Figure S16. The ^{1}H NMR (600 MHz, pyridine-*d*$_5$) spectrum of the new derivative **3c**

Figure S17. The ^{13}C NMR (150 MHz, pyridine-*d*$_5$) spectrum of the new derivative **3c**

Figure S18. The HRESIMS spectrum of the new derivative **3c**

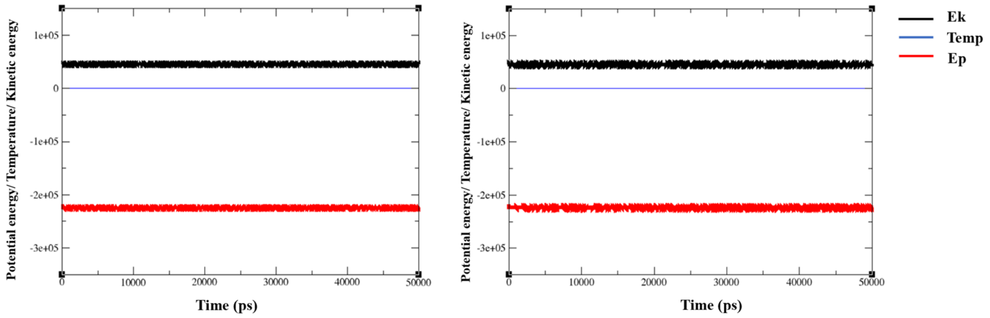

(a) **(b)**

Figure S1. Kinetic, potential energy variations and temperature changes in the MD simulations of BVM (**a**) and DSC (**b**).

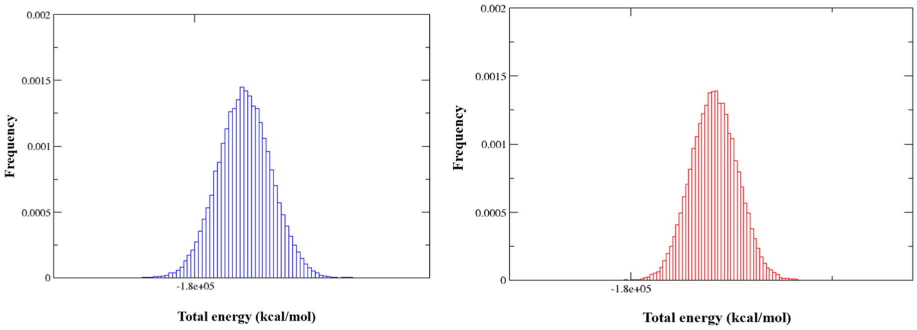

(a) **(b)**

Figure S2. Total energy frequency distribution of BVM (**a**) and DSC (**b**).

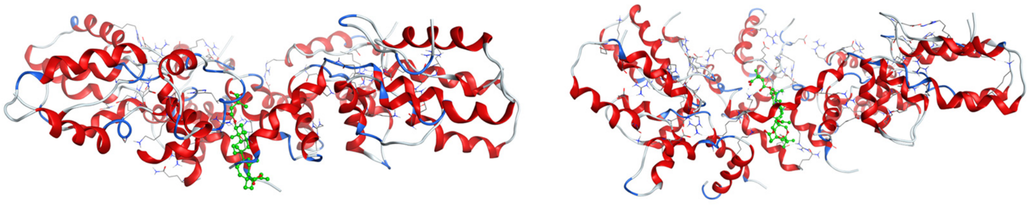

(a) **(b)**

Figure S3. Lowest potential energy structure of BVM (**a**) and DSC (**b**). The graph represented the conformation with the lowest potential energy during the MDs process, which was equivalent to the most stable conformation of the molecule and had the highest probability of occurrence in the system.

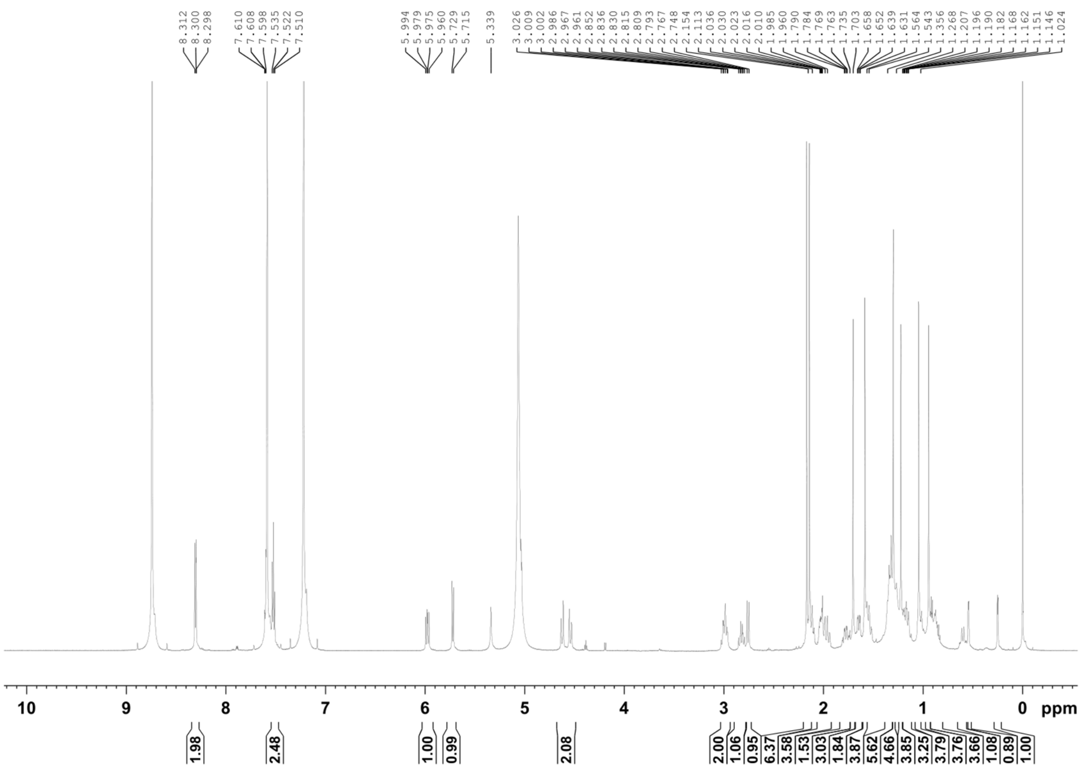

Figure S4. The ^{1}H NMR (600 MHz, pyridine-d_5) spectrum of the new derivative **2a**

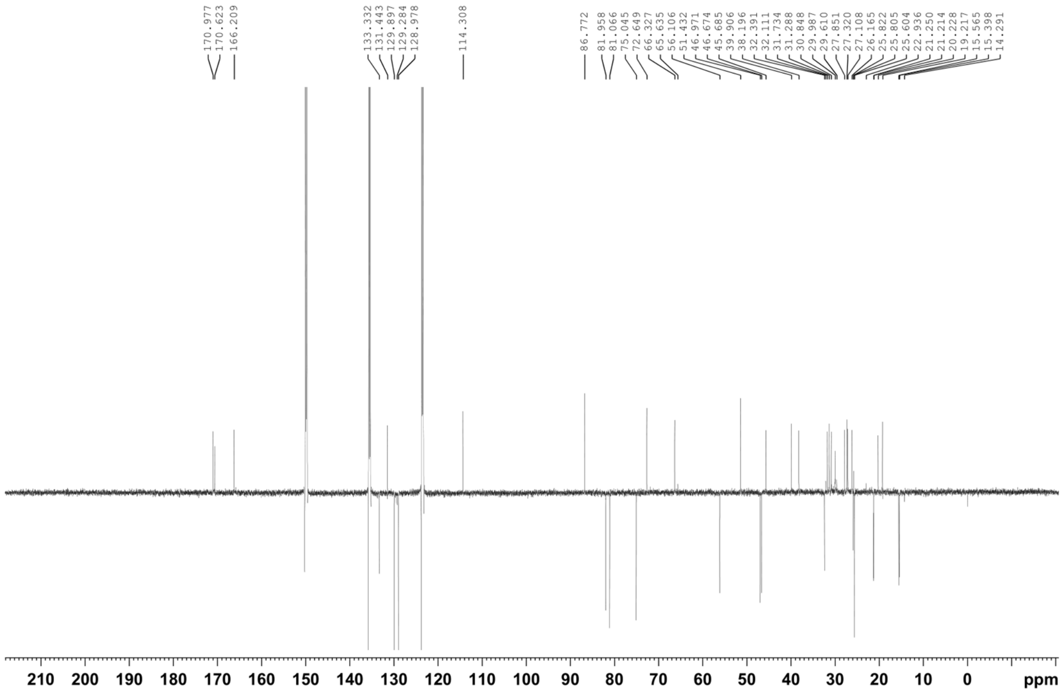

Figure S5. The ^{13}C NMR (150 MHz, pyridine-d_5) spectrum of the new derivative **2a**

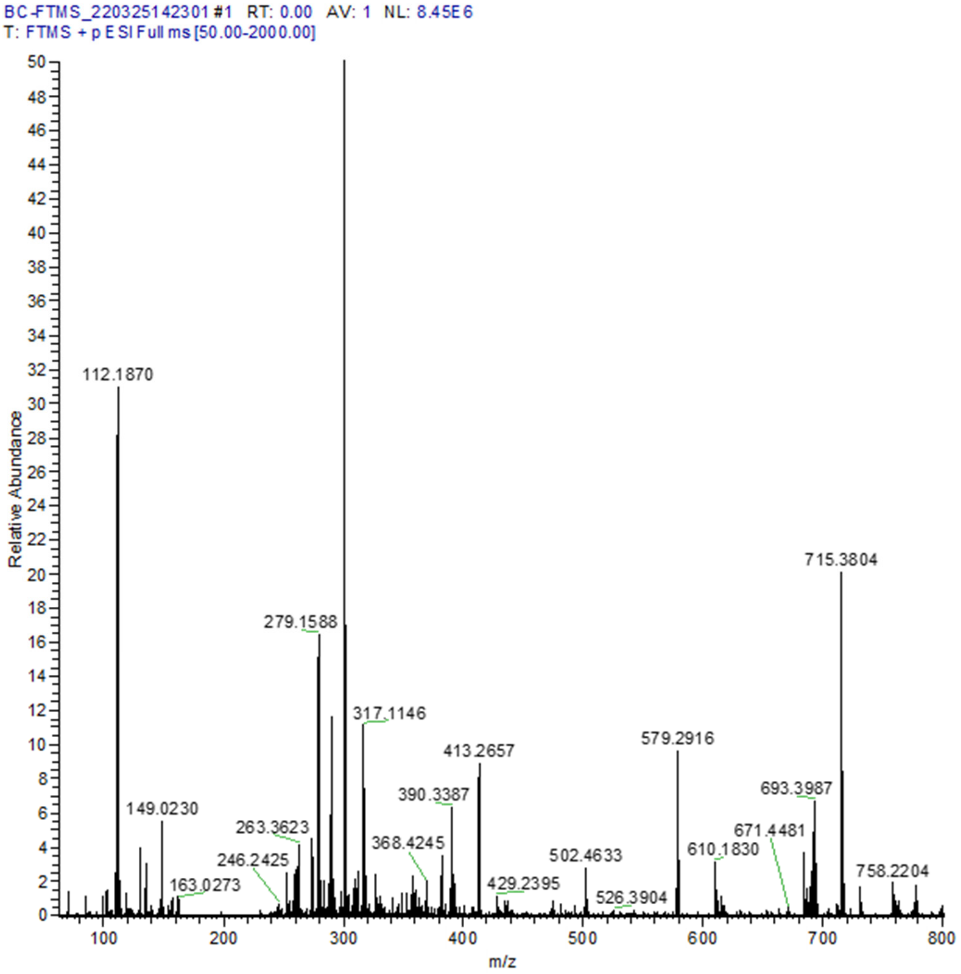

Figure S6. The HRESIMS spectrum of the new derivative **2a**

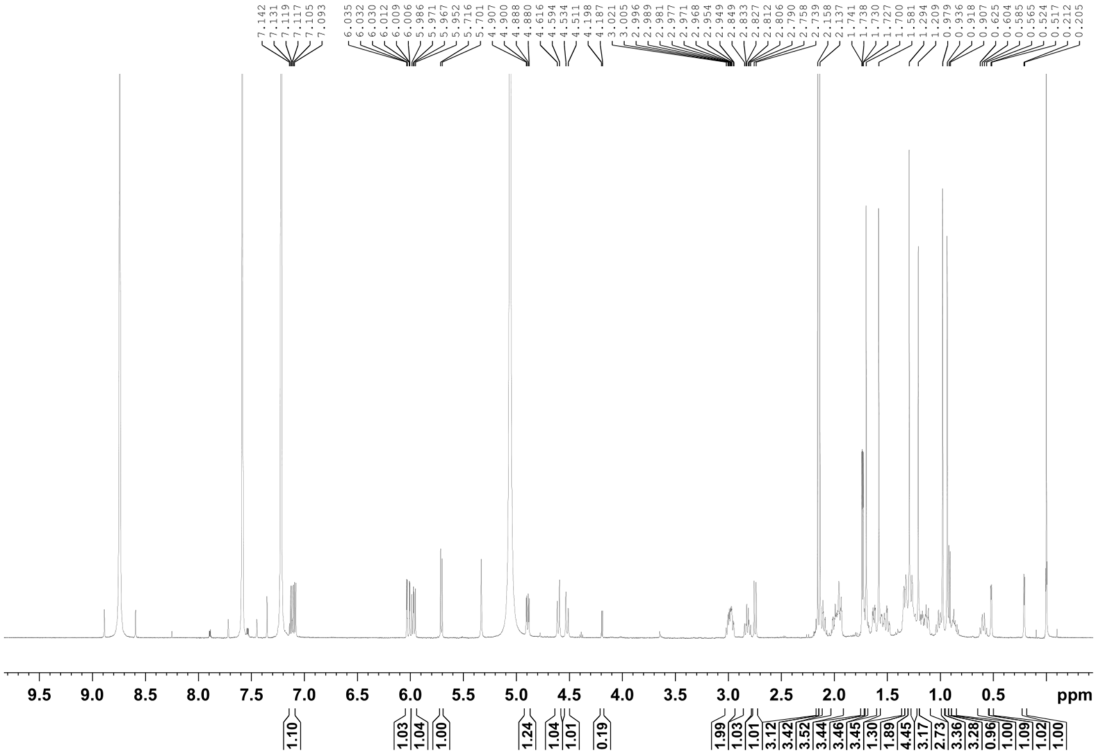

Figure S7. The ^{1}H NMR (600 MHz, pyridine-d_5) spectrum of the new derivative **2b**

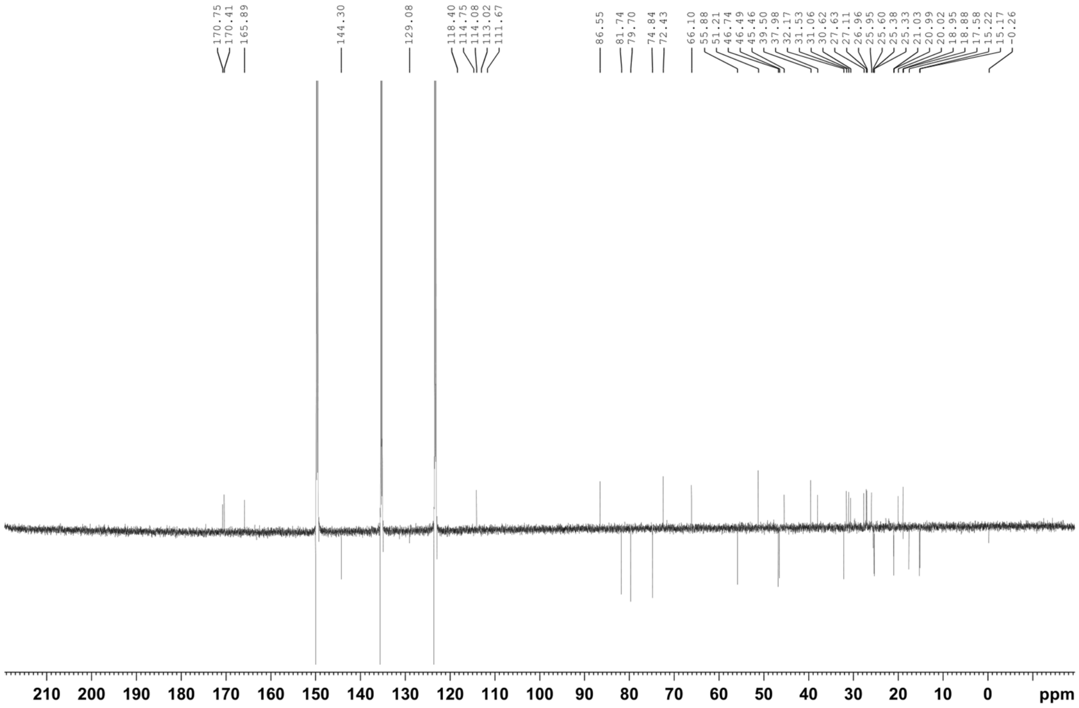

Figure S8. The ^{13}C NMR (150 MHz, pyridine-*d*5) spectrum of the new derivative **2b**

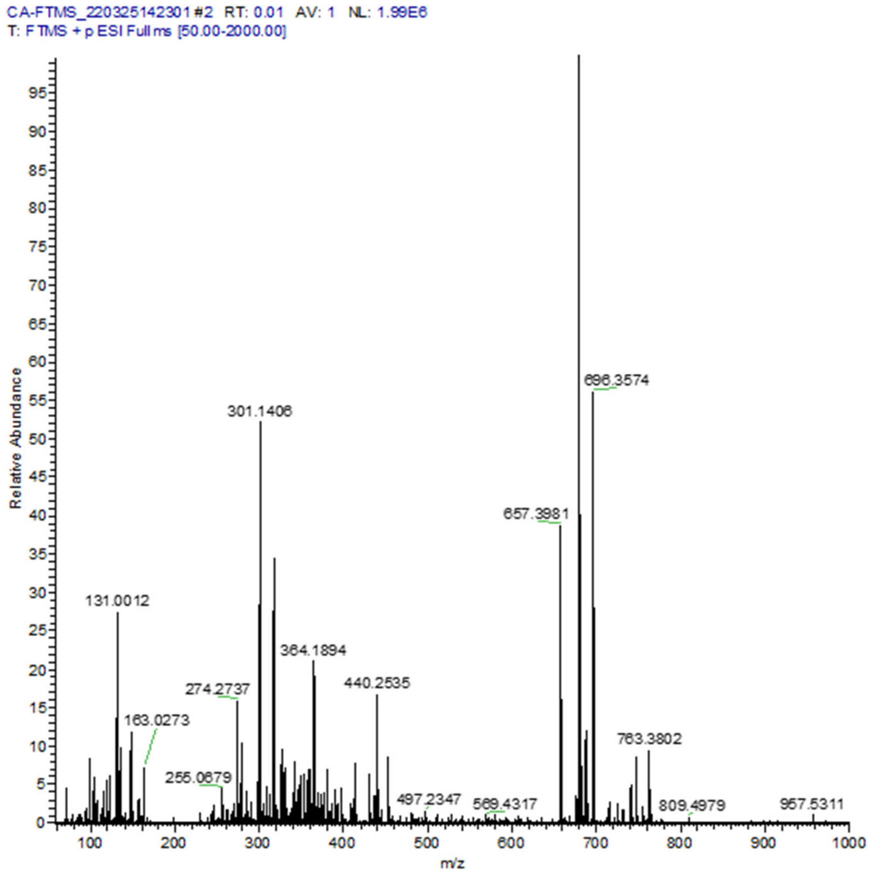

Figure S9. The HRESIMS spectrum of the new derivative **2b**

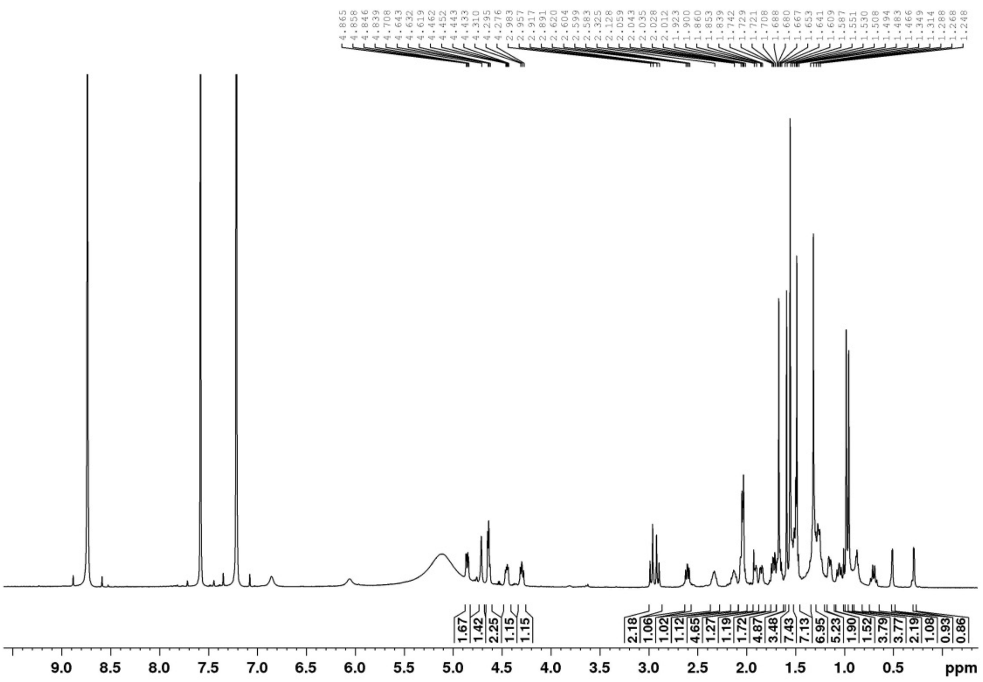

Figure S10. The ^{1}H NMR (600 MHz, pyridine-*d*5) spectrum of the new derivative **3a**

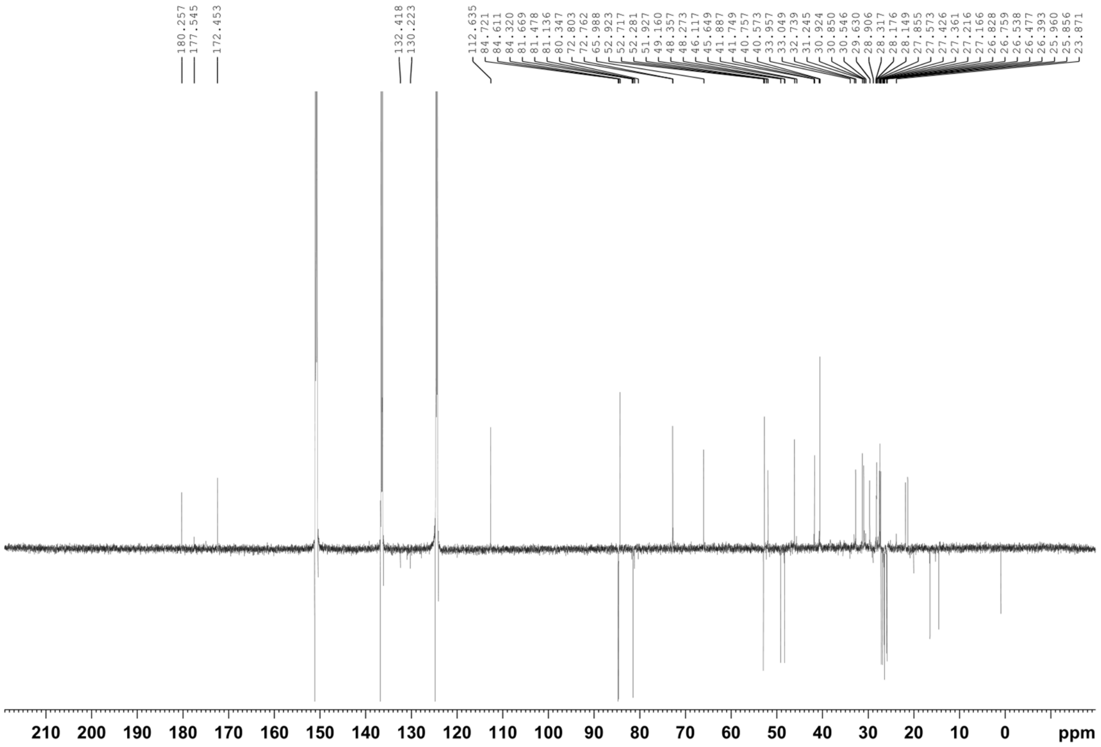

Figure S11. The ^{13}C NMR (150 MHz, pyridine-d_5) spectrum of the new derivative **3a**

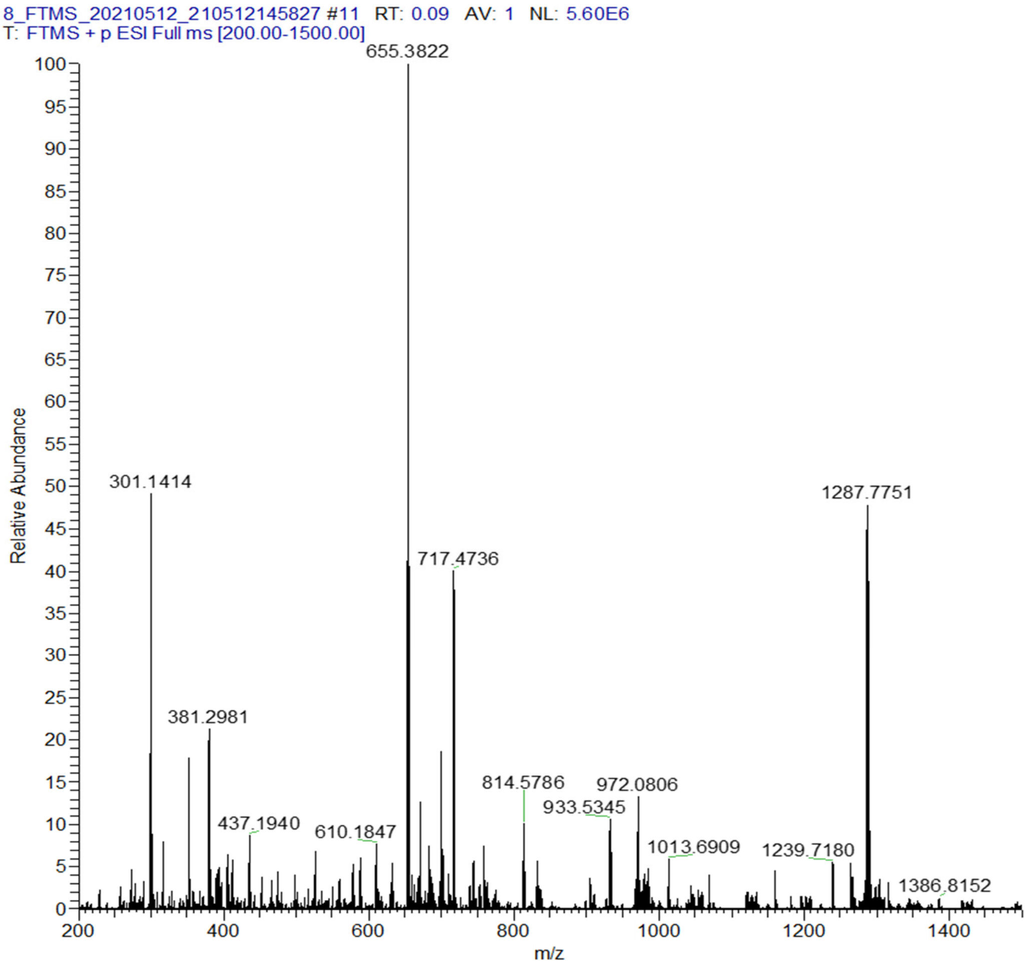

Figure S12. The HRESIMS spectrum of the new derivative **3a**

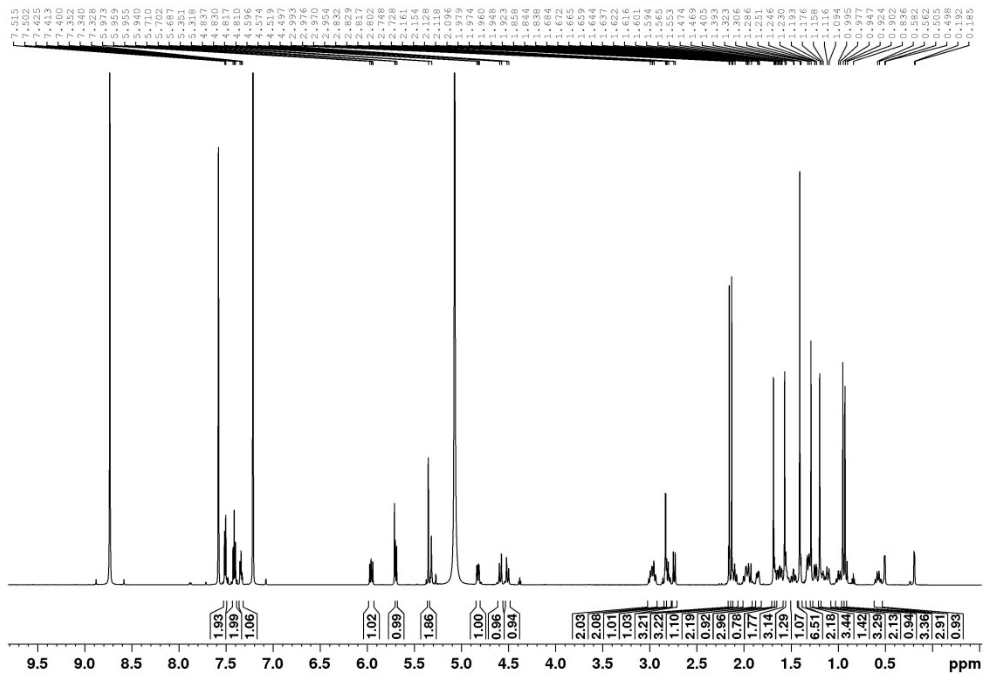

Figure S13. The ^{1}H NMR (600 MHz, pyridine-*d*5) spectrum of the new derivative **3b**

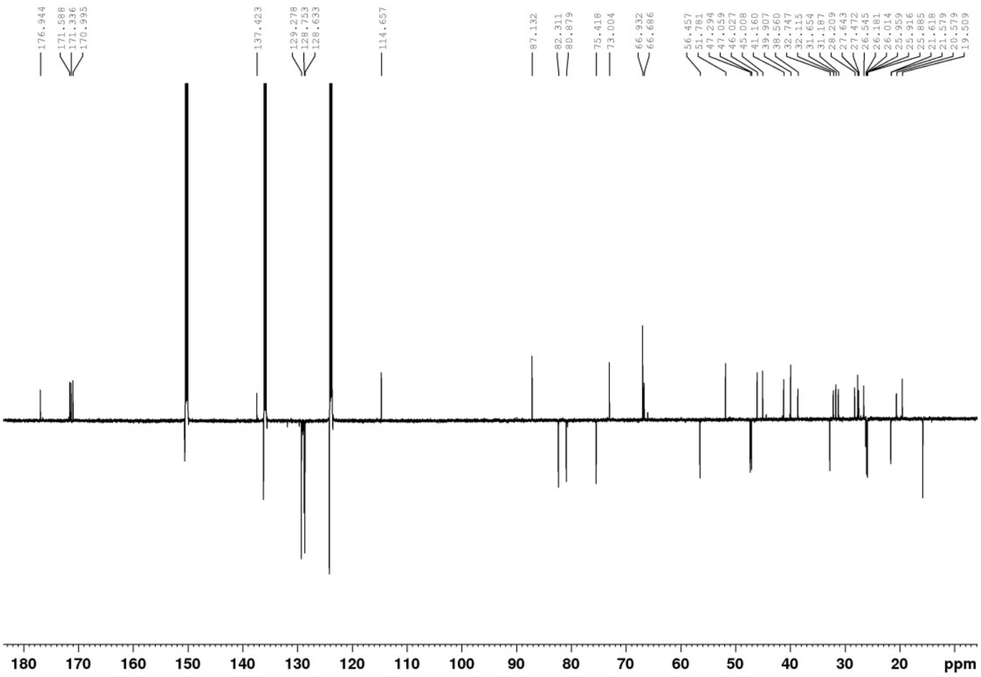

Figure S14. The ^{13}C NMR (150 MHz, pyridine-d_5) spectrum of the new derivative **3b**

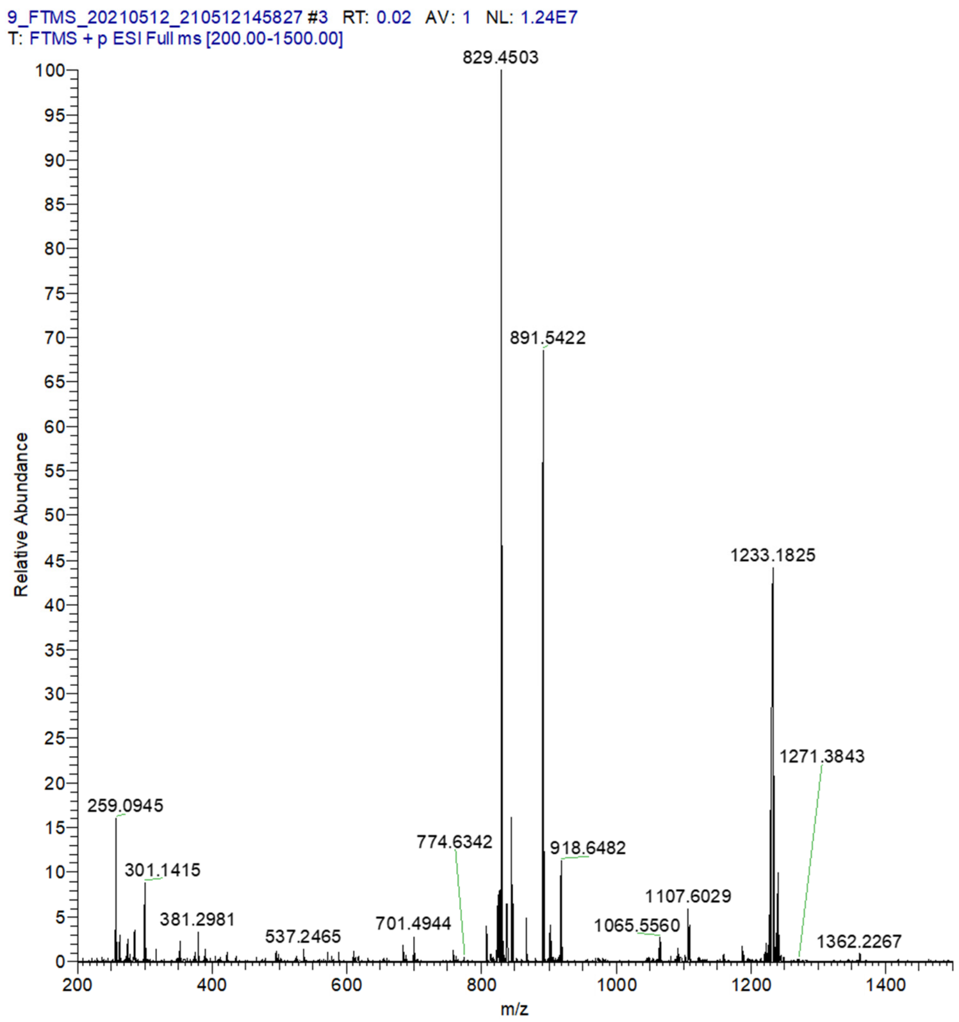

Figure S15. The HRESIMS spectrum of the new derivative **3b**

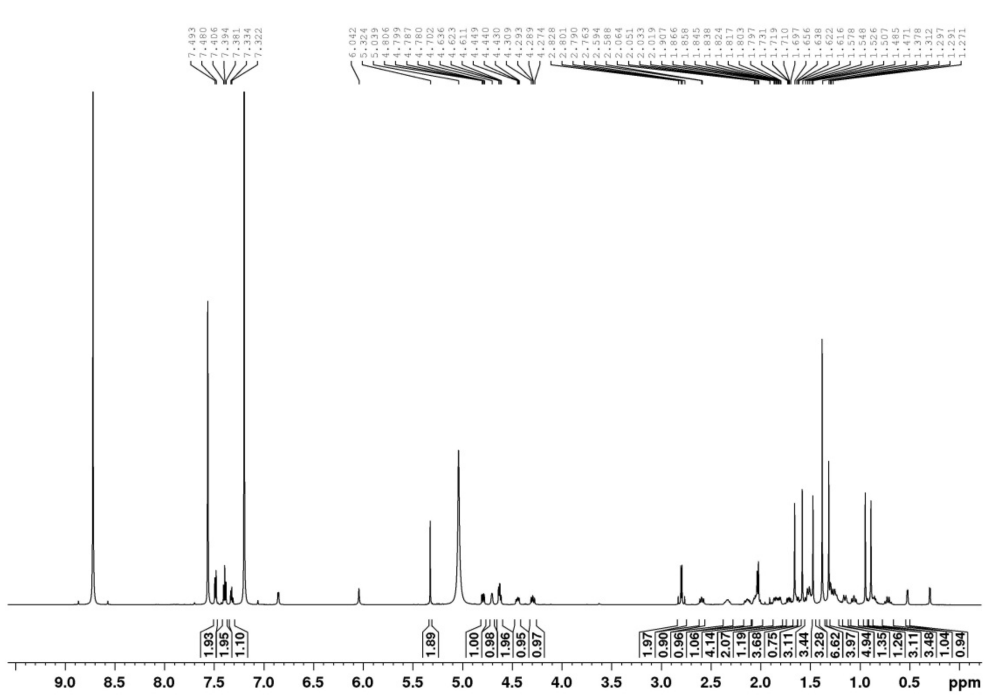

Figure S16. The ^{1}H NMR (600 MHz, pyridine-d_5) spectrum of the new derivative **3c**

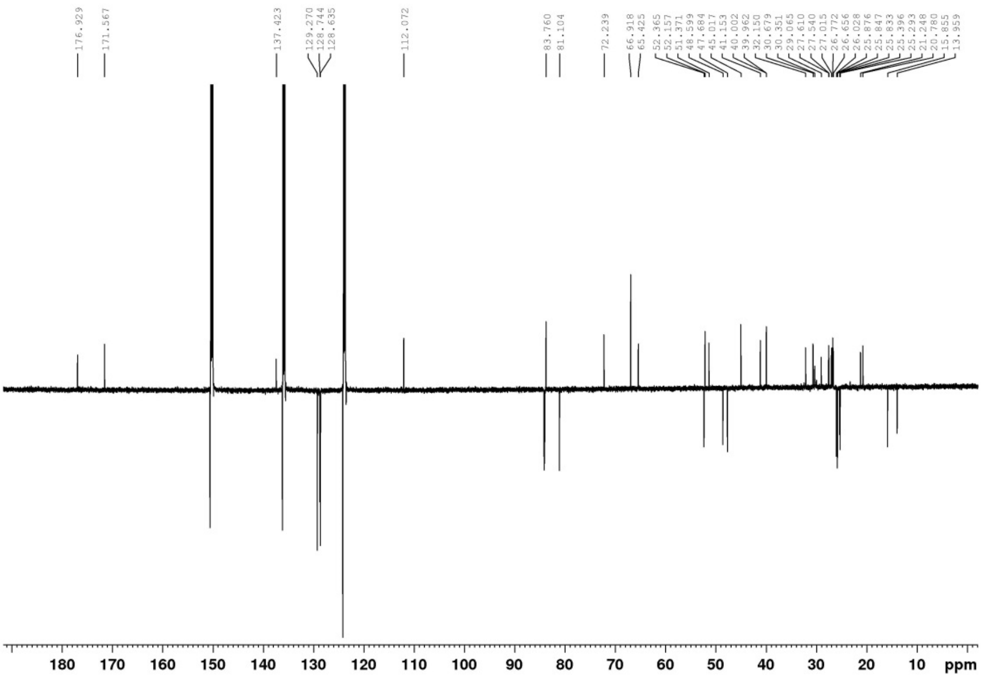

Figure S17. The ^{13}C NMR (150 MHz, pyridine-d_5) spectrum of the new derivative **3c**

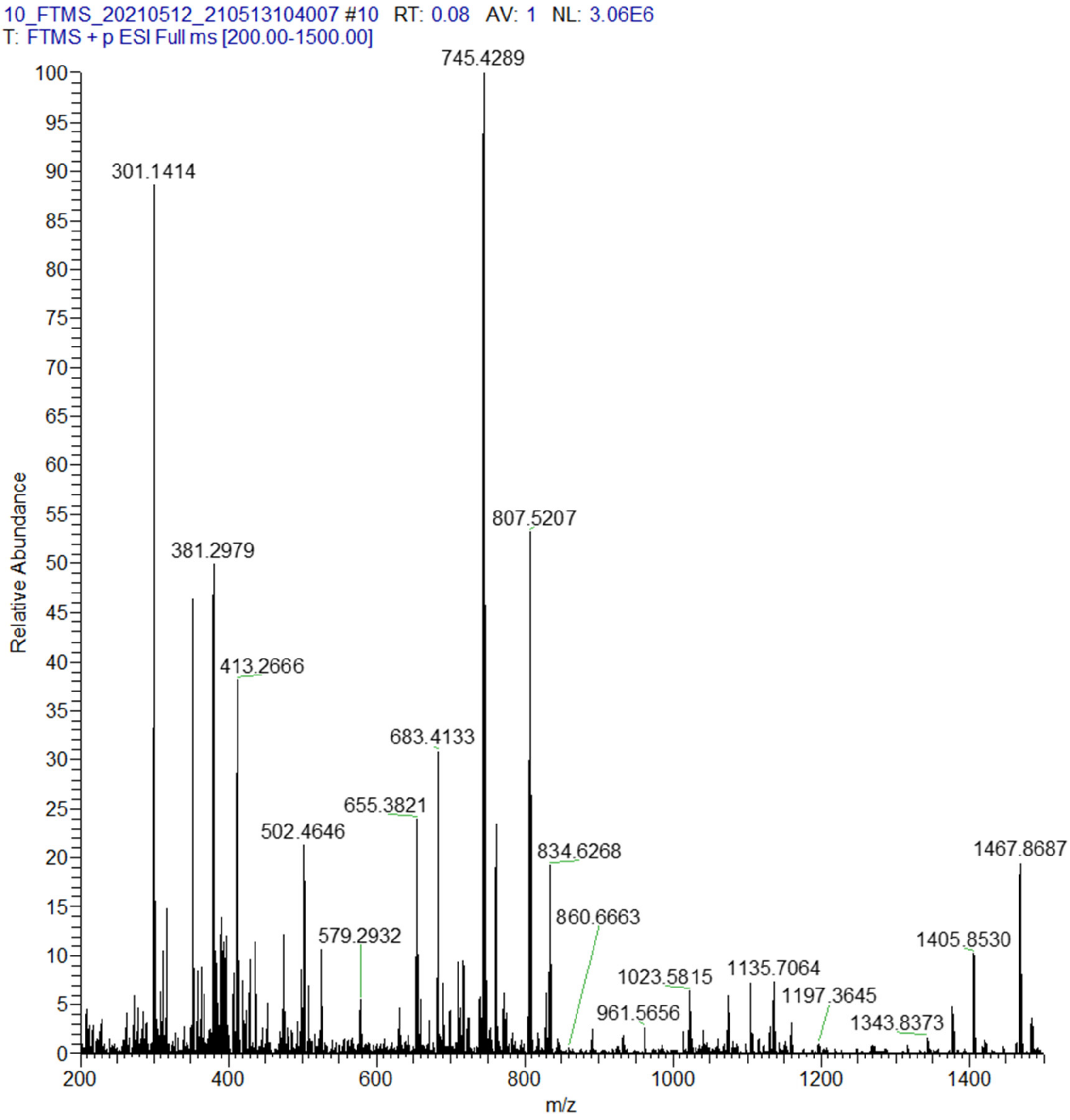

Figure S18. The HRESIMS spectrum of the new derivative **3c**
